# Mapping clinical and imaging factors that might predict cardiac events in breast cancer patients

**DOI:** 10.3389/fonc.2025.1552908

**Published:** 2025-03-11

**Authors:** Eugenia Otero-Pla, Maria Josefa Fuentes Raspall, Pedro Gallego Franco, Juan Fernández Martínez, Ignasi Gich Saladich, Nuria Jornet Sala, Maria Lizondo Gisbert, Jady Rojas Cordero, Josep Isern Verdum, Gemma Sancho-Pardo

**Affiliations:** ^1^ Department of Medicine, Universitat Autònoma de Barcelona, Barcelona, Spain; ^2^ Department of Radiation Oncology, Hospital de la Santa Creu i Sant Pau, Barcelona, Spain; ^3^ Sant Pau Biomedical Research Institute (IIB Sant Pau), Barcelona, Spain; ^4^ Department of Medical Physics, Hospital de la Santa Creu i Sant Pau, Barcelona, Spain; ^5^ Department of Cardiology, Hospital de la Santa Creu i Sant Pau, Barcelona, Spain; ^6^ CIBER Epidemiology and Public Health (CIBERESP), Hospital de la Santa Creu i Sant Pau, Barcelona, Spain; ^7^ Department of Clinical Epidemiology and Public Health, Hospital de la Santa Creu i Sant Pau, Barcelona, Spain; ^8^ Department of Radiation Oncology, Hospital Universitari de Terrassa, Barcelona, Spain

**Keywords:** cardiotoxicity, coronary artery calcium, planning CT, breast cancer, radiotherapy

## Abstract

**Background:**

Breast cancer is the most common in women, with a 90% overall survival at 5 years. Cardiotoxicity is a side effect that can modify their morbidity and mortality. Its low prevalence and long latency period have challenged the establishment of a strategy for early detection and prevention.

**Objectives:**

To investigate the association between coronary artery calcium (CAC) in planning computed tomography (CT) and cardiac events.

**Methods:**

Retrospective cohort of 873 breast cancer patients (460 right-side; 413 left-side) treated with radiotherapy (2013-2022). We extract the Hounsfield Unit to quantify the CAC degree from the heart structure in the planning CT. We used IBM-SPSS software (V 29.0 Armonk, NY) for the statistical analysis.

**Results:**

After a median follow-up of 4.52 years (range: 2.42-6.22 years), the cardiac events incidence was 1.95% vs 5.1% in right and left breast cancer, respectively. The mean heart dose was higher in cases with cardiac events (6.74Gy vs 2.28Gy; p<0.01). CAC score>0 was detected in 32.76% of planning CT and was more frequent in the elderly and those with cardiovascular risk factors (p<0.01). Patients with cardiac events presented a CAC score>0 in 41.4% of cases. However, the overall survival in these patients did not differ from those without CAC (p=0.58).

**Conclusions:**

Patients with cardiovascular risk factors and a mean cardiac dose greater than 5 Gy are at increased risk of cardiotoxicity and should be referred for Cardio-Oncology evaluation. The application of the CAC score in CT planning could be a valuable screening test that requires further study.

## 1 Introduction

Breast cancer (BC) is the leading cause of cancer in women, with overall survival of 90% at 5 years (1–3). These patients are exposed to comorbidities as the general population and additional risks from the treatments received. Cardiotoxicity is a late complication after radiotherapy (RT) that can significantly impact morbimortality (4). The earlier frequent manifestation is pericarditis, while arteriosclerosis, coronary artery disease, valvular heart disease, and arrhythmias may appear up to decades after RT (5). Epidemiological studies have observed a synergy between cardiotoxicity and RT, age over 60 years, tobacco, cardiovascular risk factors (CVRF), and history of heart disease (6, 7).

In 2013, Darby et al. (8) observed a significant relationship between RT and cardiovascular events (CVE), which increased 16% per each gray of mean heart dose (MHD) within 9 years. The MHD was higher in left breast cancer (LBC) than in right breast cancer (RBC). In clinical practice, the heart is contoured as an organ at risk, where dose distribution is heterogeneous, with the left ventricle and left anterior descending artery having higher doses in LBC versus RBC (9–14). As a result of new techniques such as intensity-modulated radiotherapy (IMRT), volumetric-modulated-arc radiotherapy (VMAT), and respiratory control, especially deep inspiration breath-hold (DIBH) technique, it is possible to reduce MHD (15–20).

In the general population aged 50-64 years, the coronary artery calcium (CAC) prevalence is 42.1% (21). Some studies have suggested that CAC has a negative predictive value for CVE. It has been observed that patients without CVRF but with a CAC score>100 Hounsfield Unit (HU) have an increased risk of developing CVE. Likewise, patients with CVRF and a high CAC have a markedly increased risk of suffering a CVE (22–24). Therefore, the relationship between CAC and CVE in cancer survivors also has been reported (25, 26). However, studies in which an association between CAC and RT has been observed have been conducted with normo-fractionation schedules. Currently, the standard of care is the hypo-fractionation schedule with excellent local-regional control without observing an increase in CVE (27, 28).

Therefore, our group has created a tool to detect the CAC in the planning CT to evaluate whether there is a relationship with CVE after RT, along with clinical and dosimetry factors. This would allow us to obtain a score to estimate cardiotoxicity risks in the future.

## 2 Methods

### 2.1 Clinical data

A retrospective review of 3.526 BC patients treated with RT between 2013 and 2022 was conducted with the approval of the Ethics Committee from our hospital (IIBSP-RAD-2023-123). Inclusion criteria were >18 years, treated with curative surgery, postoperative or preoperative RT, treated by the same highly experienced radiation oncologist, with or without systemic treatment. Exclusion criteria were bilateral BC, previous RT history, and cardiovascular disease history. Patients’ baseline data, treatments, outcomes, and cardiac events were recorded from their clinical history. We divided the stage into early and locally advanced, classifying it as locally advanced for those with T3-4N0M0 and any lymph node involvement. In addition, we grouped the following variables under cardiovascular risk factors: obesity, arterial hypertension, dyslipidemia, and diabetes mellitus. Tobacco use was collected from those patients who had used and were current smokers. The cardiac events were defined as arrhythmias, valvopathies, and coronary lesions diagnosed by electrocardiogram, echocardiography, and computed tomography coronary angiography (CTCA), respectively, in patients who reported symptoms during follow-up.

Dosimetry data were extracted from ARIA Oncology Information System and Eclipse Treatment Planning Software vs 15.6.

### 2.2 Radiotherapy treatment

The RT treatment was performed following the recommendations of the ESTRO clinical guidelines (29, 30). The patients were placed for treatment in the supine position with both arms above the head, with a Posirest-2 thoracic immobilizer (CIVCO) and respiratory control in DIBH in those cases involving the left breast. The schedules used were normo-fractionated, with 50 to 50.4 Gy in 25 to 28 fractions, and hypo-fractionated from 40 Gy to 2.67 Gy per day in 15 fractions. Tumor bed boosting was used with simultaneous integrated application up to a dose of 48 Gy to 3.2 Gy in 15 sessions, or sequentially in case of affected margins with no possibility of dose equivalent extension up to a dose of 15 Gy to 2.5 Gy in 6 fractions or 9.44 Gy to 2.36 Gy in 4 fractions.

### 2.3 CAC scoring

The CAC scoring algorithm was applied to the anatomical region, defined as the heart, during the radiotherapy treatment planning in the simulation CT scans performed on all patients. A program was created within the registry system, Application Program Interface (API), and verification was used in clinical practice (Varian Software) using the capabilities of the Eclipse Scripting Application Program Interface (ESAPI) provided by the manufacturer. The program’s objective is to analyze the structure called “heart” for each patient’s identification supplied and obtain information about the Hounsfield Units (HU) of the CAC found in that structure in the planning CT. We used the density weighting factor (DWF) utilized in the calculation of the Agatston score (23, 31) to quantify the extent of coronary artery calcification (CAC), CAC score: 130 – 199 HU (factor 1), 200 – 299 HU (factor 2), 300 – 399 HU (factor 3), > 400 HU (factor 4). The DWF is derived from the maximal CT attenuation within a given calcified lesion. A cardiology imaging specialist reviewed all the CT scans of patients with a CAC score > 0 to exclude false positives.

### 2.4 Statistical analysis

The analysis performed had a statistical significance level of 5% (α=0.05), and two-tailed tests were used. All was performed using IBM-SPSS software (V 29.0 Armonk, NY). Quantitative variables were described with mean and standard deviation, categorical variables in absolute value, and percentage. The comparison of quantitative and categorical variables was analyzed using the Student T-test and Chi-square test, respectively. The U-Mann-Whitney test was used to compare the medians in quantitative variables that did not follow a normal distribution. Multivariate logistic regression was performed to study the association between clinical parameters and dosimetry variables with CVE. The overall survival and time-to-event were calculated with Kaplan-Meier method.

## 3 Results

A total of 873 patients (LBC n=413, RBC n=460) were selected. The mean age was 61 years (20-93 years). Notably, 35.1% had a history of tobacco use, while 49% presented CVRF. Most were diagnosed in early-stage BC (84.9%) and underwent conservative surgery (81.6%), followed by normo-fractionated RT (91.1%), while 77.3% received a sequential tumor bed boost and only 27% node irradiation. RT techniques included 3DCRT (59.7%), IMRT (40.3%), and respiratory control with DIBH in LBC (43.6%). The median MHD was 2.4 (0.1-18.5) Gy. Ninety-two percent received anti-neoplastic systemic treatment, predominantly aromatase inhibitors (56.6%), anthracyclines (34.8%), tamoxifen (19.1%), and trastuzumab (14.6%) (See **Table 1**).

**Table 1:** Patient and treatment characteristics according to laterality.

	Right (N=460)	Left (N=413)	P value
Median Age	62 (20– 91)	61 (26 – 93)	<0.01
Median MHD	1.03 (0.10 – 9.86)	5.11 (0.13 – 18.5)	<0.01
Smoking habit			
No	285	282	0.56
Yes	175	131	
Cardiovascular risk factors			
Hypertension	146	126	0.69
Dyslipidemia	117	124	0.13
Diabetes mellitus	39	27	0.28
Obesity	48	44	0.92
Pathological type			
Ductal carcinoma *in situ*	30	31	0.72
Lobular carcinoma *in situ*	1	0	
Invasive ductal carcinoma	365	328	
Invasive lobular carcinoma	40	35	
Others	24	19	
Stage			
Early	402	339	0.04
Locally Advanced	58	74	
Surgery			
Lumpectomy	382	329	0.11
Mastectomy	78	84	
Systemic treatment			
Anthracycline	159	145	0.87
Trastuzumab	67	60	0.98
Tamoxifen	79	88	0.12
Aromatase inhibitors	271	223	0.14
RT technique			
3DCRT	304	217	<0.01
IMRT	156	196	
Fractionation			
Normo-fractionated	416	379	0.36
Hypo-fractionated	44	34	
Tumor bed boost			
No	98	100	0.29
Yes	362	313	
Node irradiation			
No	348	289	0.76
Yes	112	124	
DIBH			
No	460	233	<0.01
Yes	0	180	
CAC^a^			
No	304	278	0.17
Yes	147	132	

a We excluded N=12 planning CT because the script was unobtainable.

The overall survival was 93% with a median follow-up of 4.5 years (range: 2.42-6.22 years). The leading cause of mortality was BC in 50.82%, followed by secondary neoplasms (11.48%), and other causes (37.7%). No patients died of any CVE.

The CVE incidence was 3.4% at 4.5 years after RT. In the group experiencing CVE (n=30), a significant proportion was LBC (70%), had CVRF (60%), and received systemic therapy (90%). The MHD in LBC was significantly greater than RBC (p<0.01). Remarkably, the MHD of patients with CVE was higher than those without (6.74 Gy vs 2.28 Gy; p<0.01). We observed that patients treated for LBC who presented CVE (n=21) had a higher MHD compared to those without (7.90 Gy vs 4.93 Gy; p<0.01). A multivariant analysis was conducted, showing a significant association with age (OR 1.09; 95% CI 1.04 – 1.15; p<0.01) and MHD (OR 1.21, 95% CI 1.04 – 1.40; p=0.01). (See **Table 2**, **Table 3**).

**Table 2:** Bivariant analysis in patients with cardiac events.

	No cardiac events	Cardiac events	P value
Median Age	61 (52 – 69)	70.50 (59.75 – 79.25)	<0.01
Median MHD	2.28 (0.95 – 5.10)	6.74 (2.45 – 9.26)	<0.01
Side			
Right	451	9	0.01
Left	392	21	
RT technique			
3DCRT	505	17	0.73
IMRT	338	13	
Fractionation			
Normo-fractionated	767	28	0.64
Hypo-fractionated	76	2	
Tumor bed boost			
No	190	8	0.60
Yes	653	22	
Node irradiation			
No	617	20	0.44
Yes	226	10	
DIBH technique			
No	664	29	<0.01
Yes	179	1	
Smoking habit			
No	544	23	0.16
Yes	299	7	
Cardiovascular risk factors			
Hypertension	258	14	0.06
Dyslipidemia	231	10	0.47
Diabetes mellitus	63	3	0.61
Obesity	90	2	0.49
Systemic treatment			
Anthracycline	294	10	0.86
Trastuzumab	121	6	0.39
Tamoxifen	161	6	0.90
Aromatase inhibitors	478	16	0.72
CAC^a^			
No	566	16	0.14
Yes	267	12	

a We excluded N=12 planning CT because the script was unobtainable.

**Table 3:** Logistic regression of clinical and dosimetry variables for cardiac events.

	Odds Ratio	95% Confidence interval	P value
**Age**	1.09	1.04 – 1.15	<0.01
**Side**	1.64	0.45 – 5.92	0.45
**Smoking habit**	0.92	0.25 – 3.33	0.89
Cardiovascular risk factors			
Hypertension	0.71	0.18 – 2.87	0.63
Dyslipidemia	0.79	0.25 – 2.51	0.69
Diabetes mellitus	0.53	0.10 – 2.71	0.44
Obesity	0.56	0.11 – 2.88	0.49
**Fractionation scheme**	0.17	0.02 – 1.64	0.13
**Tumor bed boost**	0.71	0.23 – 2.13	0.54
**Node irradiation**	0.89	0.29 – 2.76	0.85
**DIBH technique**	0.14	0.02 – 1.23	0.08
**Mean heart dose**	1.21	1.04 – 1.40	0.02
Systemic treatments			
Anthracyclines	1.47	0.54 – 4.05	0.45
Trastuzumab	1.88	0.64 – 5.58	0.25
Tamoxifen	4.15	1.05 – 16.45	0.06
Aromatase inhibitors	1.29	0.45 – 3.73	0.64

In our cohort, the CVE predominantly consisted of valvopathies (n=14), arrhythmias (n=11), and coronary lesions (n=8). We define the group of valvopathies with stenosis and valvular insufficiency, as well as grouping all possible arrhythmias, the most prevalent being atrial fibrillation. Within the group of coronary lesions, we include the mildest to the most severe. Patients who developed valvopathies tended to be older (p<0.01), with the left side tumor (p<0.01), and had a higher MHD (7.45 Gy vs 2.32 Gy; p<0.01). Those with arrhythmias exhibited an increase in median age (p<0.01) and prevalence of CVRF (p=0.02), but none had done DIBH (p=0.03). Conversely, no statistically significant association between coronary lesions and either variable was found. We could not determine a cut-off point for MHD in our cohort to indicate an increased likelihood of a CVE.

### 3.1 Deep inspiration breath hold and radiotherapy technique

In 2017, we implemented respiratory control using DIBH in LBC (n=179), which significantly reduced MHD from 7.10 Gy to 3.23 Gy (p<0.01). Concerning the RT technique, those treated with 3DCRT presented a 1.15 Gy higher MHD than those treated with IMRT (p<0.01) (See **Table 4**). Within all patients with CVE, a single patient was treated with DIBH (p<0.01).

**Table 4:** Bivariant analysis in MHD with RT technique and laterality.

	Right	P value	Left	P value
RT technique (MHD)				
3DCRT	0.80 (0.59 – 1.23)	<0.01	4.60 (2.48 – 7.04)	<0.01
IMRT	2.32 (1.35 – 3.90)		5.60 (3.67 – 8.04)	
DIBH technique (MHD)				
No	1.03 (0.68 – 1.97)	0.3	7.10 (5-31 – 8.92)	<0.01
Yes	–		3.23 (1.86 – 4.21)	

### 3.2 Coronary arterial calcium

Based on a total of 873 planning CTs, a CAC>0 score was obtained in 42.3% of the cases, with a sensitivity of 100% and a specificity of 96% of the script applied. In 12 planning CTs the script was not executed successfully, leaving a total of 861 planning CTs in which the script was applied. Subsequently, those with a CAC score>0 (n=369) underwent a review by a cardiologist specializing in cardiac imaging, excluding 106 false positive cases due to contouring errors (22.5%), Port-A-Cath (4.9%), prosthetic valves (1.1%), and pacemakers (0.3%). The cases that were poorly contoured were delineated, and the script was reapplied, resulting in a final CAC score>0 of 32.76%. The majority had a CAC score=1 (34.62%), followed by 4 (28.32%), 2 (24.48%), and 3 (12.58%). Analysis revealed a higher prevalence of CAC involvement in RBC (n=158) than LBC (n=128).

CAC score>0 was observed in 41.4% of patients with CVE. In the bivariate analysis, we observed an increase in CAC in those at older age, CVRF (**Table 5**).

**Table 5:** Bivariate analysis of CAC score.

	CAC score =0 (N= 576)	CAC score =1 (N= 99)	CAC score =2 (N= 70)	CAC score =3 (N= 36)	CAC score =4 (N= 80)	P value
**Age (median, rang)**	57 (20 – 93)	67 (40 – 89)	68 (47 – 88)	74 (50 – 87)	77 (56 – 91)	<0.01
Smoking habit						
No	347	63	43	27	63	0.25
Yes	229	36	27	9	17	
Cardiovascular risk factors						
No	453	54	33	19	36	<0.01
Yes	123	45	37	17	44	
Systemic treatment						
No	298	35	16	12	33	0.14
Yes	278	64	54	24	47	
Cardiac events						
No	555	93	68	35	77	0.14
Yes	21	6	2	1	3	

**Figure 1:**
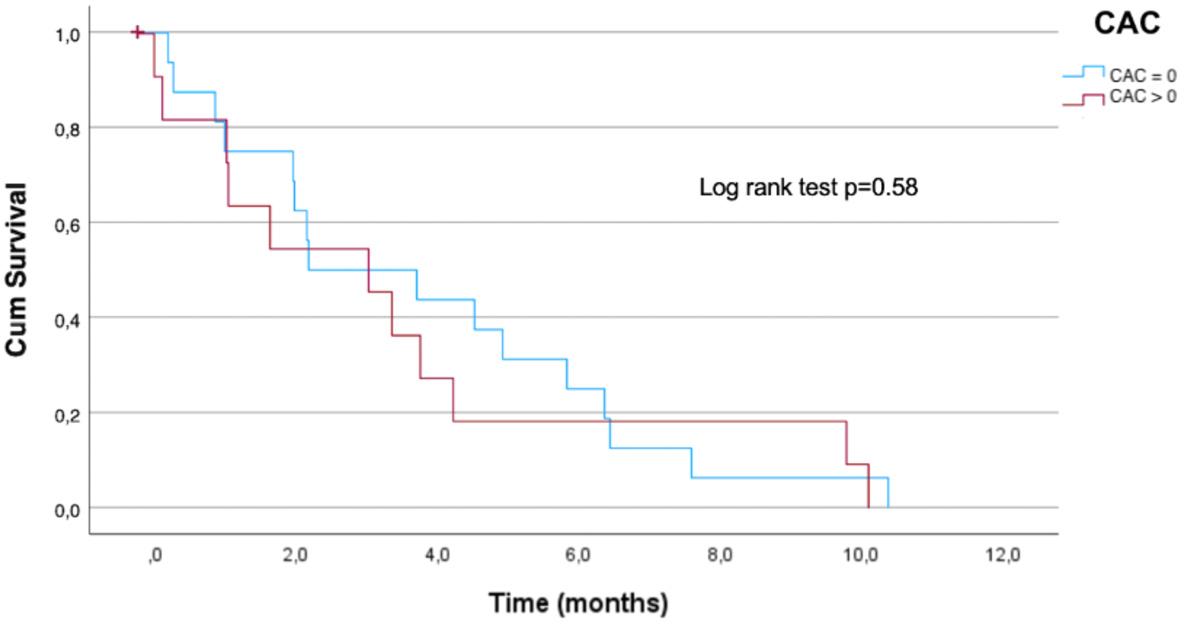
Overall survival in breast cancer patients with cardiovascular events and CAC. CAC score =0: patients who have not presented CAC in the planning CT; CAC score >0: patients who presented CAC.

Remarkably, the Kaplan-Meier method did not show an association between the overall survival and CAC score>0 in patients who had CVE (log-rank test p=0.58) (**Figure 1**).

## 4 Discussion

Radio-induced cardiac disease encompasses a wide variety of presentations that can occur during the first decade after RT. It began to be studied in the 1980s, observing a standardized CVE incidence of 1.30 at 18 years in BC survivors with an increased risk of myocardial infarction (HR= 2.55, 95% CI 1.55 to 4.19; p<0.001), valvular dysfunction (HR= 3.17, 95% CI 1.90 to 5.29; p<0.001), and congestive heart failure increased by adjuvant chemotherapy (HR= 1.85, 95% CI 1.25 to 2.73; p=0.002) (6). Once the relationship between RT and CVE was confirmed, several studies focused on risk factors for cardiotoxicity, finding a linear relationship with MHD in the fields used to irradiate the breast in the 1990s (8), as well as the predominance of events in the LBC due to cardiac anatomy, which could be decreased thanks to the DIBH technique (3–19).

The CVE incidence of our cohort was 3.4% at 4.5 years after RT, being 5.1% in LBC versus 1.95% in RBC. Therefore, our study reaffirms the correlation between CVE and MHD (p<0.01), tumor laterality (p=0.01), and the reduction of CVE in those patients who had performed the DIBH (p<0.01).

Thanks to advances, studies conducted in the 2000s (19) showed a decrease in acute coronary events incidence at 5 and 9 years of 1.9% and 3.9%, respectively, after RT, which is higher than our incidence of 0.92% at 4.5 years. This decrease was corroborated in FAST (27) and FAST-FOWARD (28) trials in which the efficacy and safety of the hypo-fractionated and ultra-hypo-fractionated scheme were evaluated, observing an ischemic heart disease incidence at 5 and 10 years of 0.9% and 1.1% respectively in the hypo-fractionated, of which 0.4% were LBC, representing 4.8% of death causes. Our study has not observed any deaths caused by CVE, probably because we have a shorter follow-up. It will be interesting to study the impact of fractionation schemes on cardiotoxicity, especially if we consider that most of our patients were treated with normo-fractionation, in contrast to the studies mentioned above that investigated the results obtained with hypo-fractionated and accelerated schemes.

Valvular heart disease is not well studied in BC patients treated with RT. Still, there are several studies in mediastinal RT treatment for Hodgkin’s lymphoma (32–34) showing a higher prevalence of valvular heart disease in those who received RT compared to those treated with single chemotherapy (12% vs. 4%, p<0.05), when they exceeded 25 Gy to the left heart related with mitral and aortic valvular disease. However, in BC patients, MHD does not usually exceed 5 Gy, and therefore, most studies focus on the most prevalent complication, such as coronary lesions. A Danish Breast Cancer Group study (35) analyzed the long-term effects, such as CVE, by contrasting treatments carried out in the non-CT and the current era. They found a 1.52% coronary heart disease incidence, 0.26% valvular heart disease, and 0.18% heart failure at 6.8 years after RT in the CT era compared to a respective 2.2%, 0.3%, and 0.23% in the non-CT era. Still, no association was found between laterality and CVE. In contrast, in our population, valvular heart disease was more prevalent than coronary lesions and was significantly related to MHD (p<0.01), laterality (p<0.01), and age (p<0.01). However, it should be noted that coronary lesions were only studied in 28 patients by CCTA, which was performed because they exhibited symptoms, and thus might be a bias for its underdiagnosis.

There are known clinical factors that predispose these patients to be more likely to have CVE after treatment (11, 36, 37). We should be aware of the following as well as thoracic RT at a young age (<50 years), the presence of CVRF, and a history of cardiopulmonary disease. One of the imaging risk factors that has begun to be studied in the general population is the CAC, particularly in those with baseline CVRF and older age, being a possible predictive tool for asymptomatic patients (21, 22). Several imaging modalities are available, but due to their cost and the risk associated with ionizing radiation, these are justified when the patient presents clinical symptoms but not for a screening and routine follow-up (23, 31, 36, 38). Hence, radiation oncologists started to study risk factors that would be able to identify a subgroup at increased risk of CVE with the planning CT (25).

In our study, we detected a CAC score>0 in 32.76% of planning CT, which was related to age (p<0.01) and CVRF (p<0.01). Although we have not found a significant association between CAC and CVE (p>0.05), we have observed that 41.4% of the patients with CVE had a CAC score>0 (p<0.01). Conversely, other authors like Roos et al. (26) demonstrated postoperative RT for BC and CAC relationship on a planning CT with acute coronary events. They had a cumulative incidence of 3.2% at 9 years, where a higher pre-treatment CAC correlated with CVE even after correcting for confounding factors such as age, heart disease history, MHD, and CVRF. However, the main limitation was that CT planning was performed in free breathing without ECG-gated. Likewise, in our study, we did not do an ECG-gated CT; we also found age and MHD as possible co-founding factors for future CVE.

In this direction, Tagami et al. (39) conducted a study where 94 women (n=49 LBC, n=45 RBC) underwent CCTA 3 years after being treated with RT between 2006 and 2019. They observed a higher coronary artery disease incidence LBC (p<0.005) and a correlation for each increased gray in MHD (p=0.03). Notably, the median MHD in this study was 1.97 (1.64-2.59) Gy, which is lower than ours, and they also found no significant relationship between CVE and smoking (p>0.9), CVRF (p=0.8) and chemotherapy (p=0.8). Although we found a significant correlation at higher MHD, we could not identify a cut-off point to discern at-risk patients. However, it is noteworthy that our results found age and CVRF as possible confounding factors due to their significant relationship with CAC by underestimating its value.

The European Cardio-Oncology guideline (40) proposed recommendations to stratify cardiotoxicity before any oncological treatment. This guideline highlights MHD as a predictive parameter for future CVE in thoracic RT treatments. Nonetheless, MHD is not the only factor to be considered in predicting the risk of cardiotoxicity. Therefore, recent studies evaluate different parameters, such as dose distribution in cardiac substructures or CAC, as other predictors (15–20).

The limitations of our study, including its retrospective nature, were the absence of ECG-gated during planning CT scans and slice thickness which led to an underestimation of CAC due to the inability to detect low calcium densities. To improve the detection of CAC, we should reduce the CT slice thickness, use the DIBH to minimize respiratory movement, and, if possible, perform ECG-gated planning CT. Finally, our brief follow-up given that radio-induced cardiac disease may appear in the first 10 years after RT, and so the sample size may need to be bigger to detect a significant number of CVE.

In conclusion, we consider that despite the lower CVE incidence, given their impact on the cancer survivors’ health, patients over 60 years with CVRF and a MHD over 5 Gy, as considered in European Cardio-Oncology guidelines, should be candidates for a Cardio-Oncology evaluation and long-term follow-up. Our findings underline the need for comprehensive risk assessment and personalized treatment strategies to mitigate it. Further research is needed to evaluate the impact of CAC on planning CT with ECG with adequate slice thickness to detect low densities and the effects of dose distribution in different cardiac cavities. A prospective study with ECG-gated planning CT to detect CAC in thoracic radiotherapy is being performed to study more accurately whether the role of CAC may be a possible predictive factor for future cardiac events.

## 5 Conclusion

Patients with CVRF and a MHD greater than 5Gy in the dosimetry plan CT should be referred for a Cardio-Oncology evaluation, given their increased risk. The application of the CAC score in CT planning could be a valuable screening test that requires further study. Further prospective studies with an ECG-gated planning CT to detect the CAC and longer follow-up are needed.

## Data Availability

The original contributions presented in the study are included in the article/supplementary material. Further inquiries can be directed to the corresponding author.
